# Pain Catastrophising Affects Cortical Responses to Viewing Pain in Others

**DOI:** 10.1371/journal.pone.0133504

**Published:** 2015-07-17

**Authors:** Nicholas Fallon, Xiaoyun Li, Andrej Stancak

**Affiliations:** Department of Psychological Sciences, Institute of Psychology, Health and Society, University of Liverpool, Liverpool, United Kingdom; University of Montreal, CANADA

## Abstract

Pain catastrophising is an exaggerated cognitive attitude implemented during pain or when thinking about pain. Catastrophising was previously associated with increased pain severity, emotional distress and disability in chronic pain patients, and is also a contributing factor in the development of neuropathic pain. To investigate the neural basis of how pain catastrophising affects pain observed in others, we acquired EEG data in groups of participants with high (High-Cat) or low (Low-Cat) pain catastrophising scores during viewing of pain scenes and graphically matched pictures not depicting imminent pain. The High-Cat group attributed greater pain to both pain and non-pain pictures. Source dipole analysis of event-related potentials during picture viewing revealed activations in the left (PHGL) and right (PHGR) paraphippocampal gyri, rostral anterior (rACC) and posterior cingulate (PCC) cortices. The late source activity (600–1100 ms) in PHGL and PCC was augmented in High-Cat, relative to Low-Cat, participants. Conversely, greater source activity was observed in the Low-Cat group during the mid-latency window (280–450 ms) in the rACC and PCC. Low-Cat subjects demonstrated a significantly stronger correlation between source activity in PCC and pain and arousal ratings in the long latency window, relative to high pain catastrophisers. Results suggest augmented activation of limbic cortex and higher order pain processing cortical regions during the late processing period in high pain catastrophisers viewing both types of pictures. This pattern of cortical activations is consistent with the distorted and magnified cognitive appraisal of pain threats in high pain catastrophisers. In contrast, high pain catastrophising individuals exhibit a diminished response during the mid-latency period when attentional and top-down resources are ascribed to observed pain.

## Introduction

Pain catastrophising has been defined as an exaggerated negative mental set brought to bear during the actual or anticipated pain experience [1,2] (reviewed in Quartana et al. [3]). In healthy people, high levels of pain catastrophising contribute to perceived pain intensity during experimental pain [1,4,5]. Pain catastrophising is also associated with increased pain severity, pain behaviour, emotional distress and disability in patients with chronic pain such as osteoarthritis [6,7], rheumatoid arthritis [8], spinal cord injury [9], fibromyalgia [10], low back pain [11,12], and musculoskeletal injury [13,14]. High pain catastrophising predicts stronger post-operative pain [15] and greater consumption of analgesics [16]. Thus, pain catastrophising contributes to both the perception of pain and to the clinical outcomes of pain [17].

The communal coping model [2,9,18,19] has been suggested as an explanatory framework for pain catastrophising. According to this model, people with higher levels of pain catastrophising communicate their pain to others to solicit social support in an attempt to mitigate their pain and pain-related emotional distress [17,20]. In support of the communal coping model, high pain catastrophisers attribute stronger pain to people exposed to a cold pressor test [21,22], and display more facial communicative pain behaviours in the presence of an observer [23]. Furthermore, high pain catastrophisers also benefit from reductions to pain intensity by disclosure of pain-related emotions [24].The attentional bias model describes pain catastrophising in terms of underlying mechanisms, as opposed to outcomes, and proposes that pain catastrophising results from an exaggerated attention to sensory and affective environmental pain cues [25]. These models are not mutually exclusive and may actually complement one another, e.g. attentional bias relates to immediate cognitive processes engaged when responding to pain-related stimuli which could necessitate social coping strategies. The present study utilises EEG to examine alterations to cortical activations which could underlie attentional bias for pain cues in high pain catastrophisers.

The neural basis of the attribution of greater pain observed in others by high pain catastrophisers is poorly understood. Functional magnetic resonance imaging (fMRI) during noxious stimulation of fibromyalgia syndrome patients revealed that patients with high pain catastrophising scores showed increased activation in the anterior cingulate cortex during pressure stimulation [26]. In healthy people, a more widespread network of regions, including parahippocampal gyrus and posterior cingulate cortex, has been shown to display greater activation during mildly painful galvanic stimulation in high–relative to low–pain catastrophisers [27]. Recently, Vase et al. [28] reported associations between pain catastrophising scores and the amplitude of mid-latency somatosensory evoked potential components originating in the secondary somatosensory cortex. Lin and colleagues [29] found a positive correlation between pain catastrophising scores and hippocampus activation during electrical stimulation of tooth pulp.

The present study expands on the previous literature by analysing the cortical activation processes underlying viewing pain in others in groups of high and low pain catastrophisers. Passive viewing of pictures depicting imminent or highly probable pain and graphically matched pictures with no imminent or probable pain were analysed using event-related potential (ERP) and source dipole analysis to evaluate spatio-temporal differences in cortical activation patterns. ERPs have been shown to differentiate pictures depicting scenes with a high risk of pain from those involving a comparatively low risk of pain [30–33]. Further, specific ERP components may be particularly relevant for pain catastrophising. The late positive potential (LPP) was previously associated with late cognitive evaluation of painful stimuli [34–36], and LPP was also proposed as a potential marker for affective regulation during aversive stimuli [37,38]. The advantages of a source analysis approach can evaluate differences in cortical activations in high and low pain catastrophisers in specific regions associated with viewing pain. Previously, these types of pictures have been shown to activate relevant brain regions including bilateral insula and anterior cingulate cortex and precuneus using fMRI [39–46].

In the present ERP study, we hypothesised that high − compared to low − pain catastrophisers would attribute stronger pain to pain scenes and manifest increased amplitudes in source activations during ERP components thought to be associated with emotional regulation or cognitive evaluation of stimuli, such as the late positive potential. By utilising source analysis we anticipate that high pain catastrophisers will demonstrate stronger activations in relevant cortical regions mediating emotional processing of observed pain.

## Materials and Methods

### Participants

Ninety-nine adult female students (aged 18−30 years) from the University of Liverpool were initially screened using the Pain Catastrophizing Scale (PCS, [1]) approximately 2 weeks prior to the experiment. All students were informed that this questionnaire concerned their thoughts and feelings when they were experiencing pain. Students were excluded if they reported a medical condition associated with pain, any neurological or psychiatric disease, or had abnormal visual ability. The average PCS score for all 99 respondents was 19.1 ± 9.8 (mean ± SD), subjects with PCS scores greater than 24 or lower than 15 were classified as high (High-Cat) and low (Low-Cat) pain catastrophisers respectively. These cut-off points represented the 66.7% and the 33.3% percentiles of all respondents. Thirty females (15 High-Cat vs. 15 Low-Cat) aged 20.3 ± 2.7 years (mean ± SD, High-Cat: 19.4 ± 1.1, Low-Cat: 21.1 ± 3.5) participated in the EEG experiment for course credits. All participants gave their informed written consent according with the Declaration of Helsinki and no minors or children were included. The study and ethics procedure was approved by the Research Ethics Committee of the University of Liverpool. All but three subjects had right-hand dominance according to self-report.

### Procedure

**Fig 1:**
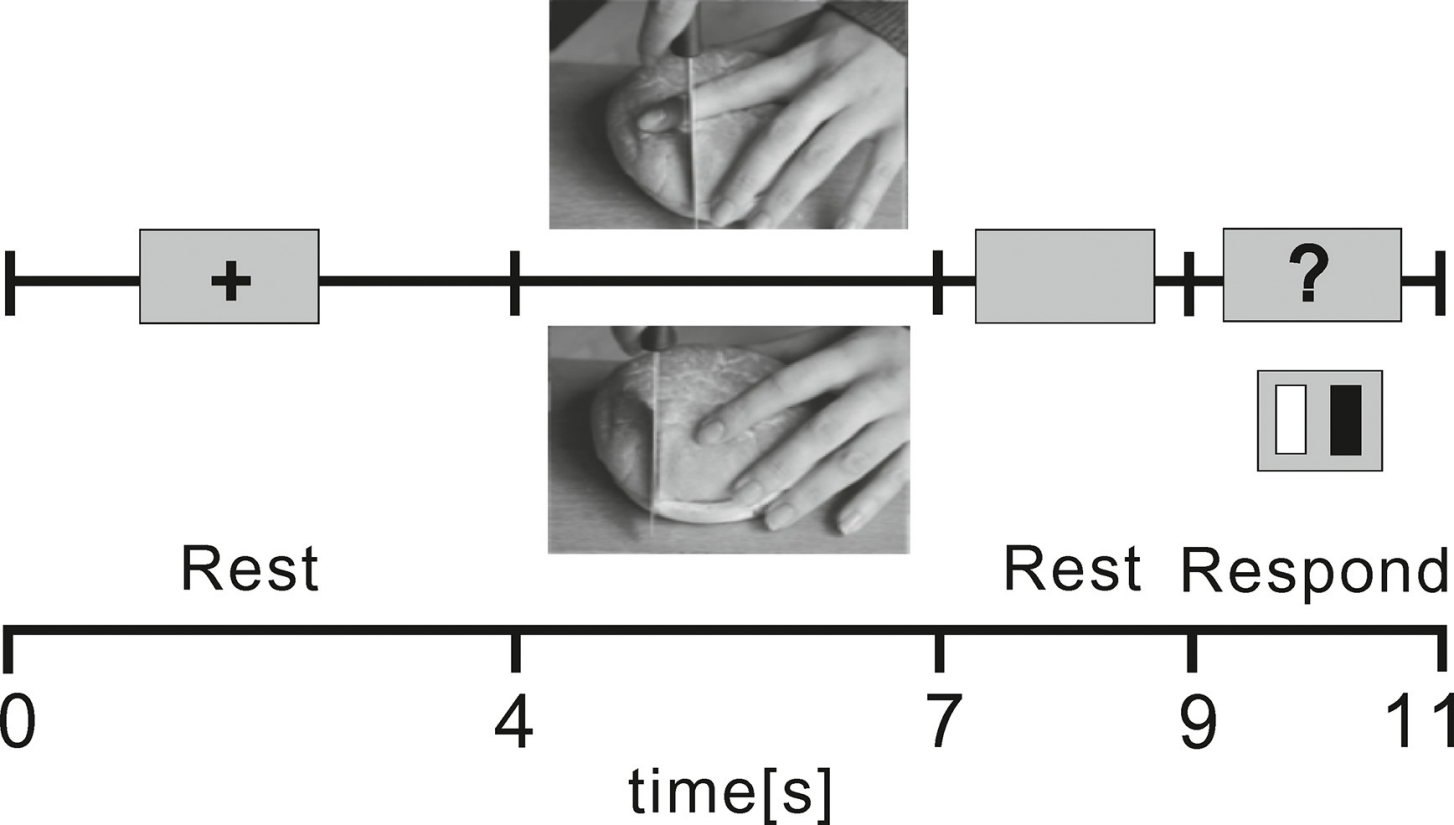
Flowchart of the experiment. The figure illustrates one trial of the experiment, beginning with a rest period (4 s) and continuing with a visual presentation of a pain or non-pain picture for 3 s, followed by another rest period of 2 s, and a response period of 2 s during which subjects pressed left or right button on a response pad to indicate whether the photograph depicted a pain or non-pain scene.

Subjects sat in a sound and light attenuated room and viewed a 19 inch LCD computer screen placed 0.7 m in front of them whilst holding a response keypad in both hands. The experiment was organised into 4 blocks each lasting 7.3 min. Each trial (Fig 1) began with a 4 s resting interval during which subjects viewed a black fixation cross on a grey background. A colour photographic image sized 425 × 319 pixels was presented on a grey background for 3 s followed by a resting interval of 2 s before a 2 s response epoch. During the response epoch, a black question mark was displayed prompting the participant to press one of two buttons to attribute whether the picture implied pain or not. The lateralisation of the button associated with pain was balanced across subjects. For synchronisation of data, the onset of pictures in the stimulus program simultaneously sent a trigger via a parallel-BNC cable interface which was recognised by EEG processing software.

In each of the four blocks, 20 pain and 20 graphically matching non-pain scenes were presented. The scenes were similar to those used in previous studies [31,39,41,42], and displayed hands or feet in situations representing implicit pain, such as a knife slicing bread in a manner that would endanger cutting the hand or syringe needle tip pressing on the skin of the forearm. Non-pain scenes were graphically matched to pain scenes but contained no potential pain, such as a knife safely cutting bread. Pairs of pain and non-pain pictures were graphically equivalent in terms of colours, contrast, objects, and viewing angles. All pictures were presented from an allocentric perspective (Fig 1). The pictures were presented at random in each block totalling 80 trials for each picture type. At the end of the experiment, subjects were instructed to rate the valence (‘‘neutral”–‘‘very unpleasant”) and arousal (‘‘neutral”–‘‘very arousing”) of each picture using 9-point Likert-style Self Assessment Manikin scales [47]. In addition, participants rated the pain attributed to each scene using a 9-point numeric scale (“no pain at all” − “worst possible pain”). Participants also completed the Interpersonal Reactivity Index (IRI,[48]) to evaluate differences in empathic behaviour between high and low pain catastrophisers. The IRI measures four scales of empathic behaviour such as empathic concern and perspective taking.

### EEG recordings

EEG data was recorded continuously using a 128-channel Geodesics EGI System (Electrical Geodesics, Inc., Eugene, Oregon, USA) with a sponge-based Geodesic Sensor Net. The sensor net was aligned with respect to three anatomical landmarks including two pre-auricular points and the nasion. The electrode-to-skin impedances were kept below 50 kΩ and at equal levels across electrodes. The recording bandpass filter was 0.1−100 Hz and the sampling rate was 250 Hz. These recording parameters were suitable for quantification of the mid-long latency ERP components which were of specific interest for the present study.

### Data pre-processing

EEG data was processed using BESA (Brain Electric Source Analysis) v. 6.0 software (MEGIS GmbH, Munich, Germany). Data were spatially transformed into reference-free data using common average reference method [49]. Oculographic and, when necessary, electrocardiographic artifacts were removed using principal component analysis [50]. Data was visually inspected for the presence of any movement or muscle artifacts, and epochs contaminated with artifacts were excluded. Event related potentials were computed separately for high and low pain catastrophisers responses to pain and non-pain trials by averaging the respective epochs in the interval ranging from 200 ms before stimulus onset to 1400 ms after stimulus onset. The baseline period ranged from -200 ms to 0 ms relative to the onset of the visual stimulus. ERP signals were bandpass-filtered from 0.5 to 40 Hz and event-related potentials from four blocks were averaged for each condition. The mean number of epochs used was 58.4 ± 10.7 for pain scenes and 59.1 ± 11.2 for non-pain scenes.

### Source dipole analysis

To evaluate differences in event-related potentials between High-cat and Low-Cat groups or picture types, and to localise the cortical regions potentially showing significant differences related to pain catastrophising, source localisations were first estimated using CLARA method (Classical LORETA Analysis Recursively Applied, [51]), as implemented in BESA v. 6.0. CLARA is a novel iterative source analysis method which operates by performing a weighted LORETA (Low Resolution Electromagnetic Tomography Analysis, [52]) on each iteration, followed by source space reduction. Compared to the standard LORETA method, CLARA reduces the blurring of the estimated sources while maintaining the advantages of a predefined distributed source model, thus making it easier to obtain a relatively focal distribution of source activation [53–55]. It combines the advantages of discrete and distributed source analysis by employing distributed source analysis with a shrinking of the source space. The default minimum regularisation cut-off parameter was used and the source image was expressed as current density within a standard MRI image (nAm/cm^3^). The ellipsoid head model was used, and the conductivities were set as follows: skin = 0.33 S/m, skull = 0.0042 S/m, cerebrospinal fluid = 1.0 S/m, and brain parenchyma = 0.33 S/m.

The source dipole model was built by applying CLARA to grand average EEG waveforms comprising all subjects and both conditions. We employed the iterative application of the LORETA algorithm to explain the potential changes occurring in the time epoch of -200 ms to 1400 ms. Four clusters were identified which exhibited activity during this interval. An equivalent source dipole was placed in the spatial maximum of each CLARA cluster and the orientation was fitted at the fixed dipole location. This four dipole solution accounted for 96% of variance in ERP data, and proved to be stable across conditions and subjects. Source locations were transformed to approximate Talairach coordinates using BESA v. 6.0.

To evaluate the effects of pain catastrophising on ERPs statistically, the grand average source dipole model was used to compute individual source waveforms during viewing of pain and non-pain pictures in High-Cat and Low-Cat groups. The source waveforms were exported by fixating the source dipole locations and refitting the orientations of all four dipoles in each subject and condition, similar to previous studies [56–59].

Data was exported to MATLAB v.7.13 (The MathWorks, Inc., USA) to analyse the average source waveforms in each of four source dipoles for pain and non-pain pictures in High-Cat and Low-Cat groups. For each source, a mixed two-way ANOVA (group × picture type) was performed across all time points to identify periods showing significant main effects or interactions. This method has the advantage of exploring the entire epoch for potential differences between conditions or groups in each source waveform, whilst controlling for the risk of type-1 error [60,61]. A 95% confidence level was employed and permutation analysis technique [62] with 2000 permutations was utilised to correct for the performance of multiple tests. Results where thresholded to only include those which covered a time window of at least 30 ms. Mean source activations were extracted from time-periods of interest for each participant in both conditions for each source for further statistical analysis using a mixed two-way ANOVA in SPSS v.20 (SPSS Inc, Chicago, USA).

### Picture ratings

Subjective ratings of valence, arousal and pain were analysed using 2×2 mixed ANOVA in SPSS. Scale values obtained from IRI in High-Cat and Low-Cat groups were compared using a Mann-Whitney U test. Spearman’s correlation coefficients were computed to evaluate the relationship between differences in source dipole components for pain and non-pain pictures and corresponding differences in subjective self-report picture ratings. To reduce the risk of type one error, Bonferrorni-Šidák’s adjustments of P values was applied. Correlation coefficients were compared between groups using Fisher’s transformation. Statistical analyses were performed using SPSS 20.0 statistical analysis package.

## Results

Table 1 shows mean PCS and IRI scores (including subscales) and output of statistical comparisons. Pain catastrophising scores were comparable to those reported in previous studies involving grouping of subjects into high and low pain catastrophising groups based on PCS scores [1,5,14,23,24,63]. High-Cat and Low-Cat groups did not differ significantly in mean IRI scores or on any of the four IRI subscales.

**Table 1:** Participant Age, Pain Catastrophizing Scale and Interpersonal Reactivity Scale scores.

	**High-Cat**	**Low-Cat**	**T(df)**	**P**	**d’**
**Age**	19.4 ± 1.1	21.1 ± 3.5	-1.85	0.08	0.7
**IRI**	70.5 ± 11.7	63.8 ± 12.0	1.56 (28)	0.13	0.59
Perspective	17.3 ± 5	17.6 ± 4.3	-1.57(28)	0.88	0.59
Fantasy	17.6 ± 7	15.6 ± 4.6	0.93(28)	0.36	0.35
Empathic concern	21.7 ± 4	19.3 ± 5.2	1.41(28)	0.17	0.53
Personal distress	13.5 ± 4.7	11.3 ± 4.5	1.37(28)	0.2	0.52
	**High-Cat**	**Low-Cat**	**U (df)**	**P**	**Z**
**PCS**	31.8 ± 5.7	9.4 ± 3.8	0 (28)	<0.001	-4.67

Age, Pain Catastrophizing Scale (PCS) and Interpersonal Reactivity (IRI) Scale scores (mean ± SD) are shown for high and low pain catastrophising groups. T values (with degrees of freedom), P values and effect sizes (Cohen’s d’) for statistical comparisons are also shown. For pain catastrophising, the output of the Mann-Whitney U test is shown.

### Picture ratings

**Fig 2:**
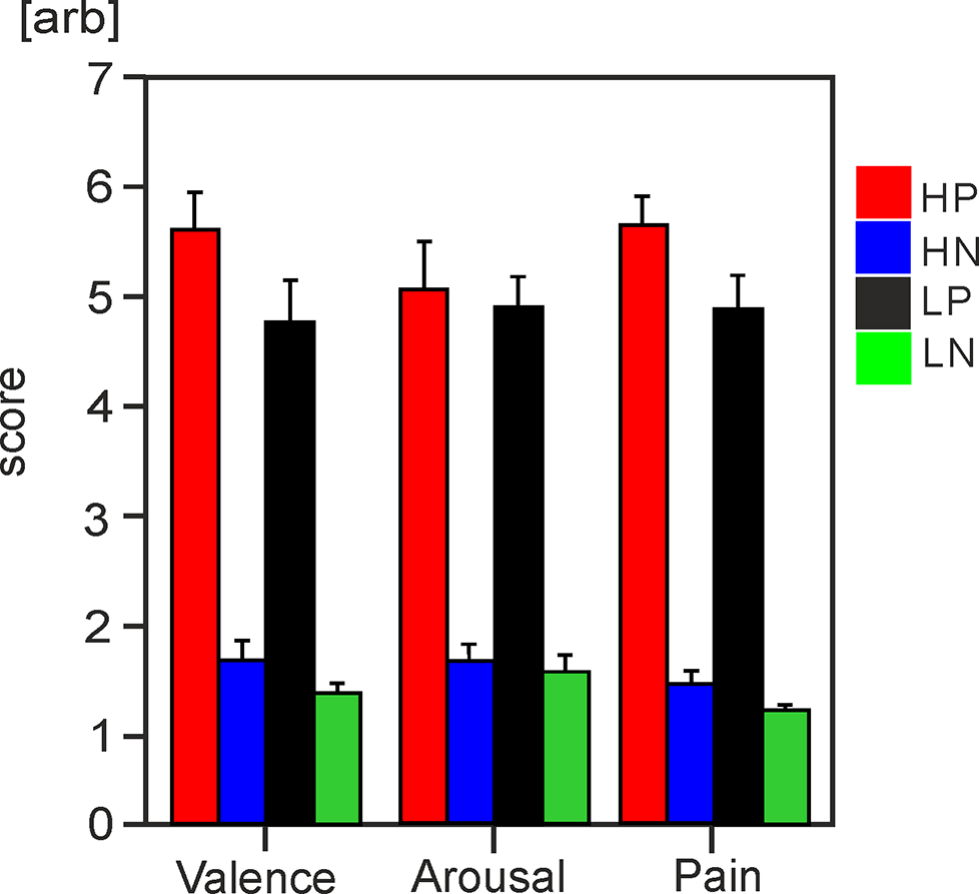
Self-report picture ratings. Bar charts with standard error bars illustrate mean values for ratings of affective valence, arousal and pain in High-Cat and Low-Cat groups for both types of pictures. HP = High-Cat pain pictures, HN = High-Cat non-pain, LP = Low-Cat pain pictures, LN = Low-Cat non-pain.

Table 2 and Fig 2 show the mean self-report values for affective valence, arousal and pain for both types of pictures in High-Cat and Low-Cat groups. For valence ratings, a two-way ANOVA for repeated measures revealed a statistically significant main effect of pain catastrophising (F(1,28) = 4.8, *P* = 0.038) with greater unpleasantness attributed to both pain and non-pain pictures by High-Cat, compared to Low-Cat, group. The main effect of picture type was also significant (F(1,28) = 196.6, *P* < 0.0001), with greater unpleasantness attributed to viewing of pain, compared to non-pain, pictures by both groups. The interaction between group and picture type was not statistically significant (F(1,28) = 0.7, *P* >0.05). Arousal ratings associated with viewing pictures were stronger for pain, than non-pain, scenes (F(1,28) = 206.5, *P* < 0.001), but similar in High-Cat and Low-Cat groups (*P* > 0.05) and the interaction effect was again not statistically significant. Participants attributed stronger pain to pain pictures compared to non-pain scenes (F(1,28) = 378.6, *P* < 0.001). High-Cat group rated both the pain and non-pain scenes as containing greater pain relative to the Low-Cat group (F(1,28) = 4.9, *P* = 0.036), and the interaction of group and picture type was not statistically significant.

**Table 2:** Valence, arousal and pain ratings attributed to pictures.

	High-Cat		Low-Cat		Effect	F(df)	P	η^2^_*p*_
	Pain	Non-pain	Pain	Non-pain				
					C	4.8	0.038	0.145
**Valence**	5.6±1.3	1.6±0.7	4.7±1.5	1.2±0.3	P	196.6	<0.001	0.880
					C*P	0.7	0.41	0.025
					C	0.28	0.604	0.010
**Arousal**	5.0±1.5	1.5±0.6	4.8±1.1	1.4±0.6	P	206.5	<0.001	0.881
					C*P	0.03	0.868	0.010
					C	4.9	0.036	0.148
**Pain**	5.7±1.1	1.3±0.5	4.9±1.2	1.1±0.1	P	378.6	<0.001	0.931
					C*P	1.83	0.187	0.061

Average ratings (mean ± SD) for valence arousal and pain attributed to pain and non-pain pictures in high, and low, pain catastrophising groups. F values (with degrees of freedom), P values and effect sizes (η^2^_*p*_) for ANOVA comparisons are also shown. C = main effect of catastrophising, P = main effect of picture type, C*I = interaction effect.

To evaluate the degree of discrimination between pain and non-pain pictures in High-Cat and Low-Cat groups, the sensitivity index (*d’*) and response bias were computed. These measures are derived from signal detection theory [64], and allow for evaluation of whether pain and non-pain visual scenes were discriminated correctly and equally in both catastrophising groups. Table 3 shows the output from signal detection analysis. One Low-Cat subject was excluded from signal detection analysis due to a missing output file. There were no statistically significant differences in hit rate, false alarm rate, sensitivity index or response bias between the two groups although response bias approached significance. Thus, High-Cat and Low-Cat groups performed similarly in discrimination of pain and non-pain scenes.

**Table 3:** Signal detection analysis.

	High-Cat	Low-Cat	T(df)	P
**Hit rate**	71.3 ± 7.3	67.93 ± 8.5	1.14(27)	0.27
**False alarm**	6.2 ± 5.5	4.7 ± 6.6	0.67 (27)	0.52
**d’**	2.9 ± 0.6	2.8 ± 0.7	0.71(27)	0.49
**Response bias**	0.01 ± 0.33	0.26 ± 0.33	2.03(27)	0.052

Output from signal detection analysis including average scores (mean ± SD) for hit rate, false alarms, sensitivity index (d’) and response bias. T values and P values from statistical comparisons of each group are also shown.

### Source dipole model

**Fig 3:**
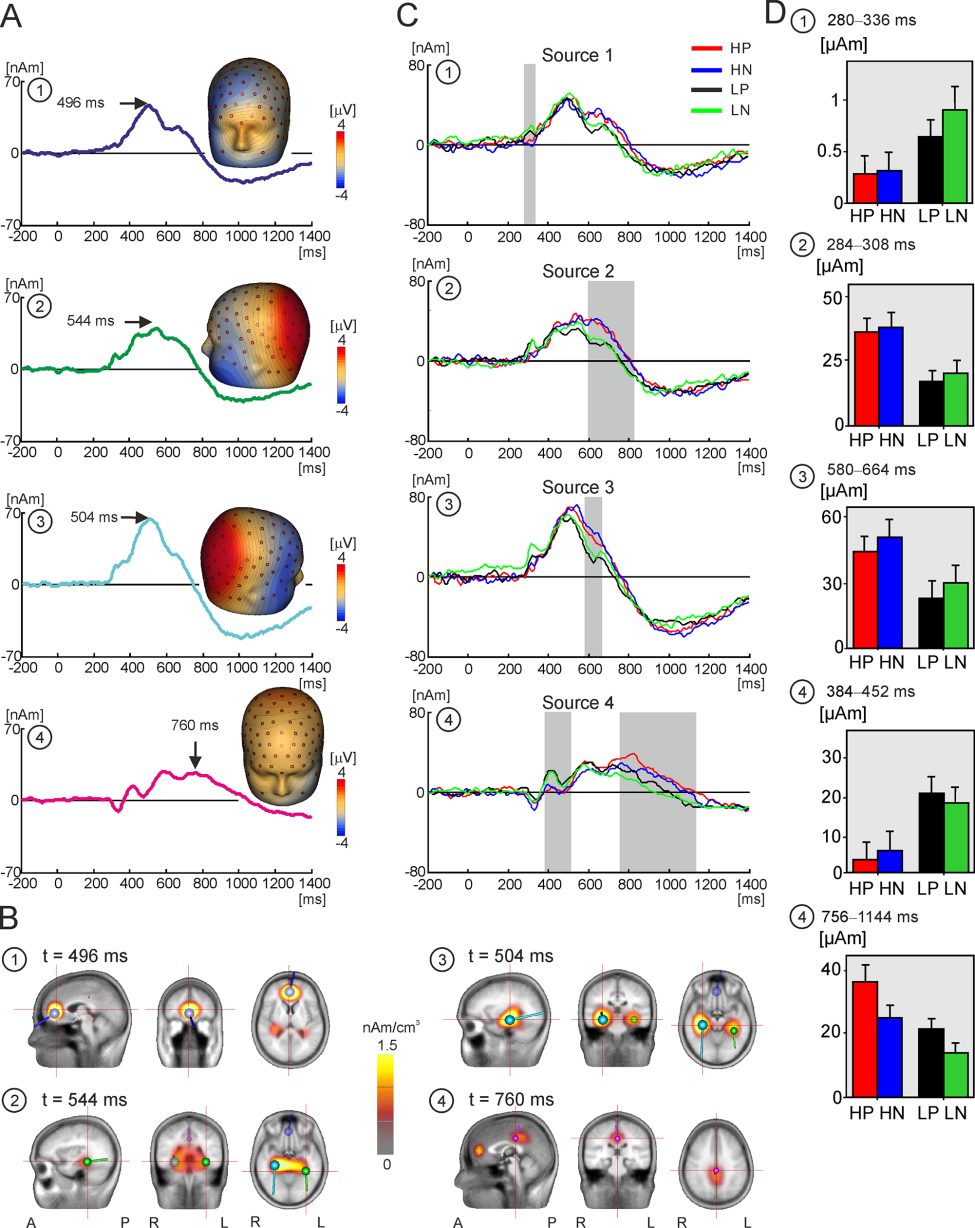
Source dipole model and source waveforms. **A.** The grand average waveforms of four equivalent source dipoles and their isopotential line maps. The isopotential maps were plotted at the temporal maxima, highlighted with an arrow and labelled with the latency value. The source dipoles are numbered from 1 to 4. **B.** CLARA source activation maps and source dipole locations of four cortical sources. The peak latency of each source corresponds to that in panel A. A = anterior, P = posterior, L = left, R = right. The numbering of dipoles corresponds to that in A. 1 = blue dipole, 2 = green dipole, 3 = ice blue dipole, 4 = magenta dipole. **C.** The grand average waveforms of four equivalent source dipoles, numbered from 1 to 4, in high and low pain catastrophising groups during viewing pain and non-pain scenes. Red line = pain photographs in High-Cat group, blue line = non-pain photographs in High-Cat group, black line = pain photographs in Low-Cat group, green line = non-pain photographs in Low-Cat group. The grey-filled rectangles indicate epochs used in statistical analyses. **D**. Bar chart of mean source activations and standard error bars for each condition during time windows of interest identified by permutation analysis (grey rectangles).

Fig 3A shows the source dipole waveforms and isopotential scalp maps of the four source dipoles across subjects and conditions. Fig 3B shows the cluster maxima and CLARA maps for each of four source dipoles. Source dipoles are numbered from 1 to 4 in Fig 3A and 3B.

Source 1 showed a maximum at 496 ms following the onset of visual stimuli. The isopotential lines, mapped at the peak of 496 ms in Fig 3A, suggested a positive maximum at the lower forehead and a negative maximum in the medial frontal region. CLARA indicated the presence of a source in the rostral anterior cingulate cortex (rACC; Brodmann area 24/32; approximate Talairach coordinates: x = 2 mm, y = 43 mm, z = 2 mm, Fig 3B). Source dipole 2 peaked at 544 ms, and accounted for the negative potential maximum seen over the left lower face and a positive potential maximum in the left posterior parietal region. This source was fitted in the left medial temporal cortex in the parahippocampal gyrus (PHG_L_; Brodmann area 36; approximate Talairach coordinates: x = -34 mm, y = -37 mm, z = -13 mm). Source 3 peaked at 504 ms, and accounted for a maximal negativity in the lower right face and a positive potential maximum in the right posterior parietal region suggesting a dipole operating in the right medial temporal cortex. Source 3 was denoted as the right parahippocampal gyrus (PHG_R_; Brodmann area 35; approximate Talairach coordinates: x = 30 mm, y = -26 mm, z = -13 mm). Finally, source dipole 4 explained the positive potential maximum at the vertex between 580 ms and 760 ms. This source showed a negativity around the chin and the neck, and the isopotential lines pointed to the presence of a dipole located deep in the medial parietal cortex. The approximate Talairach coordinates of source 4 (x = 0 mm, y = -27 mm, z = 34 mm) were consistent with a source dipole located in the posterior cingulate cortex (PCC; Brodmann area 23/31).

### Effects of pain catastrophising on source dipole waveforms

Fig 3C shows the average source waveforms in each of the four source dipoles for pain and non-pain pictures in High-Cat and Low-Cat groups. Intervals manifesting statistically significant effects of catastrophising group, picture type or interaction effects, resolved using permutation analysis across all time points, are indicated by grey rectangles. Table 4 gives the intervals used in the statistical analysis, and the mean and standard error of source dipole components for pain and non-pain pictures in each group of subjects. Fig 3D shows the mean source activations in each group and picture condition for time windows identified by permutation analysis

**Table 4:** Effects of pain catastrophising on source dipole waveforms.

Source	High-Cat		Low-Cat		Effect	F(df)	P	η^2^_*p*_
	Pain	Non-pain	Pain	Non-pain				
					C	7.18 (28)	.012	0.2
**1**	3.8±4.0	-0.5±3.7	11.7±4.0	16.0±3.7	P	<0.001	0.98	0
					C*P	1.92	0.17	0.064
					C	6.4 (28)	<0.001	0.68
**2**	36.6±5.0	37.9±5.5	16.9±5.0	20.6±5.0	P	2.47	0.127	0.081
					C*P	0.58	0.453	0.02
					C	3.95 (28)	0.057	0.124
**3**	44.0±7.3	50.4±7.6	23.4±7.3	30.7±7.6	P	5.6	0.024	0.17
					C*P	0.022	0.88	0.001
					C	6.45	<0.001	0.381
**4a**	3.6±4.5	6.0±4.7	21.3±4.5	18.9±4.7	P	0.00	1	0
					C*P	0.945	0.34	0.033
					C	5.85	0.022	0.74
**4b**	36.0±4.6	24.8±3.6	21.0±4.6	13.8±3.6	P	15.59	<0.001	0.36
					C*P	0.711	0.41	0.025

Source dipole moments (mean ± SD, nAm) in select time epochs in high and low pain catastrophisers during viewing pain or non-pain pictures. F values (with degrees of freedom), P values and effect sizes (η^2^_*p*_) for ANOVA comparisons are also shown. C = main effect of catastrophising, P = main effect of picture type, C*I = interaction effect. Source 1 = rostral anterior cingulate cortex 280–336 ms; Source 2 = left paraphippocampal gyrus 596–828 ms; Source 3 = right parahippocampal gyrus 580–664 ms; Source 4a-4b = posterior cingulate cortex 384–452 ms and 756–1144 ms respectively.

In source 1, located in the rACC, source activity was stronger in Low-Cat, relative to High-Cat, group for both types of pictures in the time epoch 280–336 ms after stimulus onset (Fig 3C and Table 4). Between 596–828 ms, Source 2, located in PHG_L_, demonstrated a statistically significant main effect of group. The effect was due to stronger source activity in the High-Cat, compared to Low-Cat, participants. In the time interval 580–664 ms, amplitude of source 3, located in PHG_R_, was stronger during viewing of non-pain than pain pictures.

In source 4, fitted in the PCC, a statistically significant effect of pain catastrophising was shown in the latency epoch of 384 ms to 452 ms, hereafter referred to as source 4a, which was caused by a larger source amplitude in Low-Cat than High-Cat group. In contrast, source activation in the late interval (756–1144 ms, hereafter referred to as source 4b the long latency (756 ms) was stronger in the High-Cat, relative to Low-Cat, group. The main effect of picture type was also significant in this time interval with pain pictures eliciting stronger source activity relative to non-pain pictures.

### Correlations between source components and picture ratings

**Fig 4:**
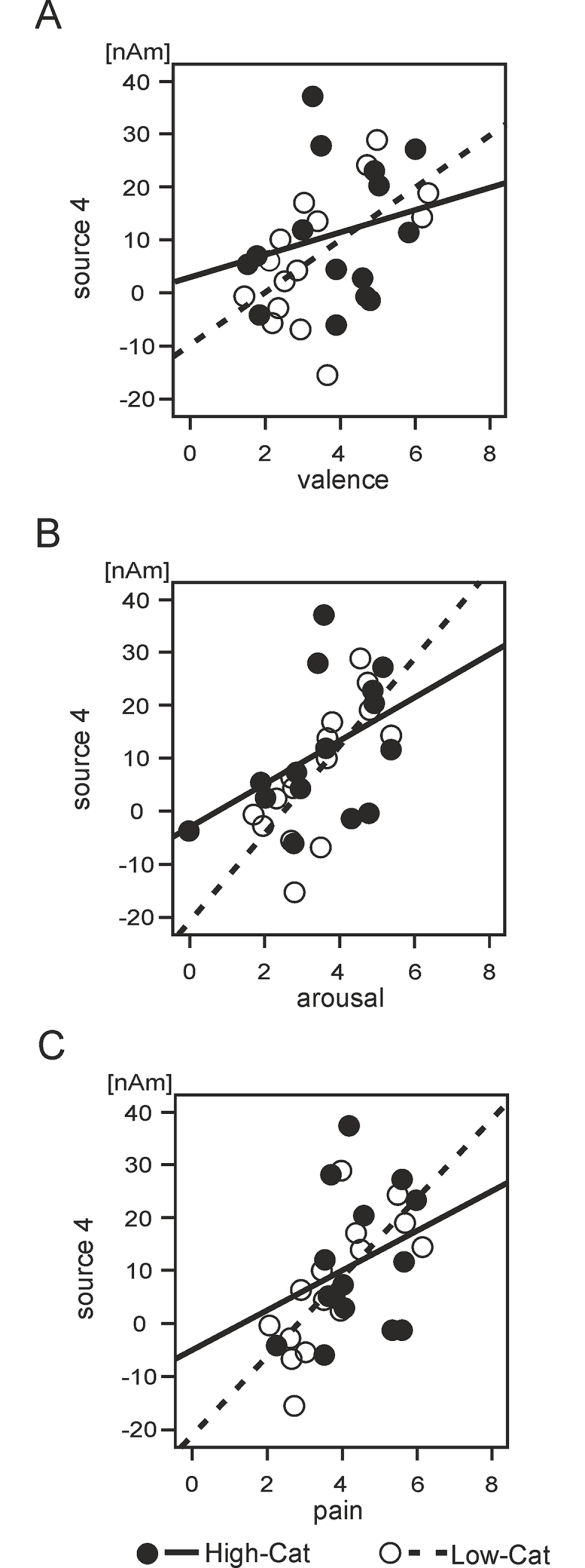
Correlations between source components and picture ratings. Scatter plots and the linear regression lines illustrating relationships between subjective ratings of valence, arousal and pain attributed to visual stimuli and the source amplitude differences between two conditions in the posterior cingulate source dipole in the interval of from 756 to 1144 ms. **A.** Valence. **B.** Arousal. **C.** Pain. High-Cat = high pain catastrophisers, dark circles, solid line. Low-Cat = low pain catastrophisers, white circles, dashed line.

Spearman’s correlation coefficients were calculated between the source activation differences [pain–non-pain pictures] in sources and intervals manifesting statistically significant effects of group (Table 4) and the [pain–non-pain] differences in subjective ratings of valence, arousal and pain. Table 5 shows Spearman’s Rho correlation coefficients and statistical values for bivariate correlations between each source activation and subjective ratings of valence, arousal and pain. We found two statistically significant correlation coefficients surpassing Bonferroni-Šidák corrected P values between the amplitude of the long latency (756–1144 ms) PCC source (source 4b) and arousal and pain ratings in the Low-Cat group only. Fig 4 illustrates the scatter plots and linear regression lines for the valence, arousal and pain rating scales and the source amplitude differences of the late PCC activation in High-Cat and Low-Cat groups. Results suggest that the activation of the PCC during the late latency epoch in the Low-Cat group demonstrates a significantly stronger relationship with subjective arousal and observed pain elicited by visual stimuli than in the High-Cat group. Correlations between source activations and between the respective picture ratings can be found in supplementary materials S1A and S1B Table.

**Table 5:** Correlations between sources activations and subjective ratings.

**High-Cat**						
**Source**	**Valency**		**Arousal**		**Pain**	
	rho	P	rho	P	rho	P
**1**	0.03	0.92	0.35	0.2	0.16	0.58
**2**	0.34	0.21	0.3	0.28	0.44	0.10
**3**	-0.45	0.88	0.79	0.78	-0.24	0.39
**4a**	-0.12	0.68	0.01	0.97	-0.09	0.74
**4b**	0.17	0.55	0.49	0.064	0.34	0.22
**Low-Cat**						
**Source**	**Valency**		**Arousal**		**Pain**	
	rho	P	rho	P	rho	P
**1**	0.30	0.27	0.38	0.17	0.41	0.13
**2**	-0.21	0.45	-0.14	0.62	-0.4	0.14
**3**	0.08	0.79	-0.13	0.63	0.09	0.76
**4a**	0.37	0.18	0.36	0.19	0.37	0.18
**4b**	0.58	0.023	0.74	0.002*	0.8	<0.001*

Spearman’s correlations (Rho and P values) for the difference between pain and non-pain pictures in source activations and subjective ratings of valence, arousal and pain. Correlation is significant at the P<0.05 level (two-tailed) following Bonferroni-Šidák correction for multiple tests.

## Discussion

High pain catastrophising individuals attributed more pain to pictures depicting high, and even low, risk of pain being inflicted. The comparison of cortical activations during passive viewing of pictures revealed several differences between High-Cat and Low-Cat groups. High-Cat participants exhibited a blunting of the cortical response in the rACC and PCC between 280 to 450 ms after stimulus onset, which was followed by a pattern of augmented cortical responses in PHG_L_ and PCC during the late latency window (> 600 ms). Furthermore, only the Low-Cat group demonstrated a correspondence between source strength of the PCC activation and pain and arousal ratings during the late latency period. This correlation was weaker in the High-Cat group, suggesting an undifferentiated and amplified response during the late period of processing pain and non-pain pictures in High-Cat participants.

### Picture ratings

Participants with high pain catastrophising scores attributed greater pain and unpleasantness to visual scenes depicting somatic pain in others than participants with low pain catastrophising scores, which accords with previous research [17,20]. Further, the High-Cat group also attributed more pain to pictures depicting a low risk of pain, which supports the stipulation that high pain catastrophising individuals are more likely to interpret stimuli as painful even if there is only a remote, indirect or small possibility of pain being inflicted [25,65]. This generalised sensitivity to somatic visual stimuli was evident despite the fact that discrimination between pain and non-pain pictures was appropriate and similar for both High-Cat and Low-Cat groups. Our findings support the attentional bias theory of pain catastrophising which argues that pain experience is amplified via exaggerated attention to sensory and affective cues [25], and accord with empirical evidence pointing towards attention bias as a cognitive mechanism for pain catastrophising behaviour (reviewed in Quartana et al. [3]).

### Mid-latency ERPs

In accordance with previous neuroimaging studies of observed pain [41–46], we found evidence for rACC, bilateral PHG and PCC involvement in processing of pain and non-pain pictures in both high and low pain catastrophising participants, with a significantly stronger activation during pain pictures evident in the PCC. The mid-latency window (280‒450 ms) demonstrated a positive potential with a spatial maxima over central-parietal electrodes, corresponding with the latency and topographic location of the P300 component. The amplitudes of source activations in the rACC and PCC were reduced during this period in High-Cat, compared to Low-Cat, participants for both types of stimuli. P300 amplitude is known to reflect the influence of top-down processes such as attentional factors, task relevance, stimuli salience and motivational significance during passive viewing of emotional stimuli [37,38,66,67] as well as subjective arousal [38]. Previous studies have found that phobic participants exhibit an enhanced P300 to fear related pictures due to the extreme motivational salience of stimuli [68–70]. Similarly, our research group has recently shown that chronic pain patients with fibromyalgia syndrome manifest augmented P300 responses to both pain and non-pain pictures, suggesting increased salience and attention for somatic stimuli with even a low risk of pain [71]. By contrast, experienced physicians viewing pain pictures demonstrate reduced P300 amplitudes and subjective pain ratings for observed pain, due to desensitisation to pain-related stimuli [30]. Likewise, the present study shows that High-Cat, compared to Low-Cat, participants manifest attenuated P300 amplitudes for both types of stimuli in the rACC and PCC. This would suggest that the appraisal of attentional allocation, stimuli salience and motivational significance for both pain, and non-pain, pictures is altered in the High-Cat group. Alternatively, mid-latency ERP components have also been associated with emotional regulation [37], and activations for both types of stimuli may reflect self-regulatory processes to control affective responses to the pictures. One can speculate that such preventative regulation may be lacking in High-Cat participants, leading them to eventually over-react to any somatic stimuli, which could explain the augmented late activation profile.

A previously meta-analyses of fMRI studies of passive viewing of pain identified ACC as a core region for empathic processing [46]. Furthermore, both anterior and posterior cingulate cortices have been proposed as neural generators contributing to P300 salience-related responses (reviewed in Linden, [72]). Our findings of reduced cortical activations in these regions suggest that high pain catastrophising individuals exhibit blunted processing of visual scenes with somatic content during the mid-latency window which is important for appropriate allocation of motivational significance, attention to stimuli and other top-down influences on processing of observed pain.

### Long-latency ERPs

In contrast to the mid-latency findings, the long latency window (600 ‒1100 ms) demonstrated stronger cortical activations in High-Cat, relative to Low-Cat, group in the PHG_L_ and PCC. This long-latency window corresponds to the late positive potential (LPP), which is evoked by passive viewing of emotional stimuli [73–76], and characterised by a positive potential located over central-parietal regions of the scalp [66]. The LPP was previously associated with cognitive evaluation of painful stimuli [34–36]. Increased LPP amplitudes may also indicate emotional regulation during viewing of affective pictures, particularly during aversive stimuli [74–77]. In accordance with previous findings [31,78], our study reveals that LPP responses, generated in the PCC, were enhanced in both groups for pain, relative to non-pain, pictures. Furthermore, the stronger LPP activations in PCC and PHG_L_ in High-Cat participants accord with previous findings of augmented LPP amplitudes elicited by threatening/ feared stimuli in a high anxiety [79], and phobic participants [69].

Consistent with a recent source localisation study of phobic patients undergoing exposure to fear stimuli [80], the present study revealed that the LPP response to pain and non-pain pictures was generated in the PCC and medial-temporal cortices. In humans, the PCC is engaged during emotion regulation [81] and episodic memory retrieval [82], amongst various other functions (reviewed by Vogt and Laureys, [83]). The enhanced LPP amplitude in these regions raises the possibility that High-Cat participants require greater resources for emotional regulation when observing somatic scenes, or are more likely to relate pain (and even non-pain) cues to prior pain experience. This finding may relate to observed difficulties in disengaging from pain cues in High-Cat individuals [63,84]. Furthermore, the results show that the magnitude of PCC activation in this time period demonstrated a significantly stronger positive correlation with arousal and subjective pain ratings of stimuli in Low-Cat, relative to High-Cat, participants. In high pain catastrophising participants, the late latency activation in the PCC was augmented for both types of stimuli, but did not correspond to levels of observed pain or arousal. We can speculate that the augmented activations relate to a generalised state of arousal for somatic cues in High-Cat participants, although further research is needed to corroborate this. LPP amplitude was previously, proposed to reflect subjective arousal associated with processing of affective stimuli [38,66,85].

The late latency activations in the present study were seen in bilateral medial temporal cortices encompassing hippocampus, parahippocampal gyri and entorhinal cortex. Previously, functional lateralisation of the medial temporal cortices was proposed, with the right side usually activated first by emotional stimuli, and mediating a global emotional reaction, with the left hemisphere being more engaged in cognitive-emotional processing of stimuli [86,87]. In support of this explanation, a previous ERP study for observed pain reported predominantly left hemisphere activation differences when differentiating painful and neutral pictures [31]. The present study similarly identified independent activation profiles for each of the medial temporal cortices. The activation in the PHG_R_ peaked 40 ms prior to PHG_L_, and was stronger for non-pain pictures across both groups, whereas the PHG_L_ displayed stronger activations in High-Cat, relative to Low-Cat, participants and covered a significantly larger time window. These findings suggest that the left medial-temporal cortex was more likely to contribute to altered evaluation of pictures in High-Cat participants.

The study of extreme groups based on PCS scores introduces some inherent limitations concerning interpretation of findings. Although we reveal a neurophysiological difference between high and low pain catastrophisers, it is not possible to accurately infer whether either group would exhibit specific differences relative to normal PCS respondents. Thus, future research using a larger cohort and including a full range of respondents would be beneficial. Similarly, the tertile cut-offs used to define groups based on PCS scores means that few group members may be included as high or low catastrophisers despite scoring relatively normal scores. This limitation of the group descriptors and the method should be considered when interpreting the findings. On the other hand, the same factor has the added benefit of improving the relevance of findings for respondents with less extreme PCS scores. It is also possible that the responses to non-pain pictures could be in some way influenced by the pain context of the experiment, and the presence of pain cues in 50% of trials. This could also explain the finding of enhanced mid-latency activations to both types of stimuli in Low-Cat participants, with augmented activation profiles seen later in High-Cat group. This limitation should be considered in future research, e.g. it may be useful to include a further comparison with no somatic content.

To sum up, the findings indicate that individuals with a high pain catastrophising trait initially demonstrate a pattern of blunting of the cortical response during early appraisal of pain and non-pain pictures, before an augmented pattern of cortical activation is established during the late period of cognitive evaluation of stimuli. The cortical activation differences may reflect alterations to the initial appraisal of stimuli salience and motivational significance, followed by a greater allocation of resources for emotional regulation, or an enhanced engagement with painful (and even non-painful, somatic) cues in high pain catastrophising individuals. The findings may be indicative of generalised sensitivity to somatic pictures in High-Cat individuals, although the contrasting directions of mid and long latency source activation differences points towards a complex mechanism underlying the augmented subjective ratings for pain and non-pain pictures. Pain catastrophising contributes to perceived pain intensity during experimental pain [1,4,5], and it is also associated with increased pain severity, emotional distress and disability in chronic pain patients with osteoarthritis [6,7], rheumatoid arthritis [8], fibromyalgia [10] and low back pain [11,12]. Thus, pain catastrophising can be considered as a predictor for a variety of pain-related outcomes, both in healthy and chronic pain populations [3]. By improving our understanding of the mechanisms by which catastrophising influences the pain experience, we can begin to unlock the potential clinical benefits of addressing pain catastrophising itself.

## Supporting Information

S1 Table Correlations between source activations and picture ratings. A. Spearman’s correlations (Rho and P values) for the activation difference between pain and non-pain pictures in each of the five source activations. *Correlation is significant at the P<0.05 level (two-tailed) following Bonferroni-Šidák correction for multiple tests. B. Spearman’s correlations for the activation difference between pain and non-pain pictures for pain, valence and arousal rating scales.
(DOCX)
