# Supplementary material for: Pain Catastrophising Affects Cortical Responses to Viewing Pain in Others
Source: PLoS One. 2015 Jul 17;10(7):e0133504. doi: 10.1371/journal.pone.0133504 (PMC4505849; doi:10.1371/journal.pone.0133504)
Supplement: S1 Table — A. Spearman’s correlations (Rho and P values) for the activation difference between pain and non-pain pictures in each of the five source activations. *Correlation is significant at the P<0.05 level (two-tailed) following Bonferroni-Šidák correction for multiple tests. B. Spearman’s correlations for the activation difference between pain and non-pain pictures for pain, valence and arousal rating scales. (DOCX) [file pone.0133504.s001.docx]

**S1 Table A. Correlations between source activations and picture ratings**

| **High-Cat** | | | | | | | | | | | | |
| --- | --- | --- | --- | --- | --- | --- | --- | --- | --- | --- | --- | --- |
| **Source** | **1** | | **2** | | | | **3** | | **4a** | | **4b** | |
|  | rho | p | rho | | p | | rho | p | rho | p | rho | p |
| **1** |  |  | 0.35 | | 0.2 | | 0.23 | 0.42 | 0.64 | 0.011 | 0.029 | 0.92 |
| **2** | 0.35 | 0.21 |  | |  | | 0.09 | 0.73 | 0.55 | 0.035 | -0.42 | 0.12 |
| **3** | 0.23 | 0.42 | 0.09 | | 0.73 | |  |  | 0.45 | 0.10 | -0.17 | 0.54 |
| **4a** | 0.64 | 0.011 | 0.55 | | 0.035 | | 0.45 | 0.10 |  |  | -0.38 | 0.16 |
| **4b** | 0.03 | 0.92 | -0.42 | | 0.12 | | -0.17 | 0.54 | -0.38 | 0.16 |  |  |
| **Low-Cat** | | | | | | | | | | | | |
| **Source** | **1** | | | **2** | | **3** | | | **4a** | | **4b** | |
|  | rho | p | rho | | p | | rho | p | rho | p | rho | p |
| **1** |  |  | -0.28 | | 0.31 | | 0.13 | 0.65 | 0.74 | 0.002* | 0.29 | 0.28 |
| **2** | -0.28 | 0.31 |  | |  | | -0.25 | 0.37 | -0.2.6 | 0.34 | -0.36 | 0.19 |
| **3** | 0.13 | 0.65 | -0.25 | | 0.37 | |  |  | 0.30 | 0.27 | -0.15 | 0.59 |
| **4a** | 0.74 | 0.002* | -0.26 | | 0.34 | | 0.30 | 0.27 |  |  | 0.31 | 0.26 |
| **4b** | 0.30 | 0.28 | -0.36 | | 0.19 | | -0.15 | 0.56 | 0.31 | 0.26 |  |  |

**B.**

| **High-Cat** | | | | | | | | |  |
| --- | --- | --- | --- | --- | --- | --- | --- | --- | --- |
| **Scale** | **Valence** | | **Arousal** | | | | **Pain** | |  |
|  | rho | p | rho | | p | | rho | p |  |
| **Valence** |  |  | 0.82 | | <0.001* | | 0.8 | <0.001* |  |
| **Arousal** | 0.82 | <0.001* |  | |  | | 0.8 | <0.001* |  |
| **Pain** | 0.8 | <0.001* | 0.8 | | <0.001* | |  |  |  |
| **Low-Cat** | | | | | | | | | |
| **Source** | **Valence** | | | **Arousal** | | **Pain** | | |  |
|  | rho | p | rho | | p | | rho | p |  |
| **Valence** |  |  | 0.88 | | <0.001* | | 0.77 | <0.001* |  |
| **Arousal** | 0.88 | <0.001* |  | |  | | 0.83 | <0.001* |  |
| **Pain** | 0.77 | <0.001* | 0.83 | | <0.001* | |  |  |  |

**A.** Spearman’s correlations (Rho and P values) for the activation difference between pain and non-pain pictures in each of the five source activations. **B.** Spearman’s correlations for the activation difference between pain and non-pain pictures for pain, valence and arousal rating scales. *Correlation is significant at the P<0.05 level (two-tailed) following Bonferroni-Šidák correction for multiple tests.
